# Protein Nanoparticles as Vaccine Platforms for Human and Zoonotic Viruses

**DOI:** 10.3390/v16060936

**Published:** 2024-06-09

**Authors:** Kush K. Pandey, Bikash R. Sahoo, Asit K. Pattnaik

**Affiliations:** 1 School of Veterinary Medicine and Biomedical Sciences, University of Nebraska-Lincoln, Lincoln, NE 68583, USA; kpandey2@huskers.unl.edu (K.K.P.); bsahoo2@unl.edu (B.R.S.); 2 Nebraska Center for Virology, University of Nebraska-Lincoln, Lincoln, NE 68583, USA

**Keywords:** protein nanoparticles, viral vaccines, ferritin, lumazine synthase, encapsulin, computationally designed nanoparticles

## Abstract

Vaccines are one of the most effective medical interventions, playing a pivotal role in treating infectious diseases. Although traditional vaccines comprise killed, inactivated, or live-attenuated pathogens that have resulted in protective immune responses, the negative consequences of their administration have been well appreciated. Modern vaccines have evolved to contain purified antigenic subunits, epitopes, or antigen-encoding mRNAs, rendering them relatively safe. However, reduced humoral and cellular responses pose major challenges to these subunit vaccines. Protein nanoparticle (PNP)-based vaccines have garnered substantial interest in recent years for their ability to present a repetitive array of antigens for improving immunogenicity and enhancing protective responses. Discovery and characterisation of naturally occurring PNPs from various living organisms such as bacteria, archaea, viruses, insects, and eukaryotes, as well as computationally designed structures and approaches to link antigens to the PNPs, have paved the way for unprecedented advances in the field of vaccine technology. In this review, we focus on some of the widely used naturally occurring and optimally designed PNPs for their suitability as promising vaccine platforms for displaying native-like antigens from human viral pathogens for protective immune responses. Such platforms hold great promise in combating emerging and re-emerging infectious viral diseases and enhancing vaccine efficacy and safety.

## 1. Introduction

The earliest report of vaccination dates to over 250 years ago when Thomas Dimsdale introduced powdered scabby pustules containing variola virus into the arm of Catherine the Great, the empress of Russia, to protect her from smallpox. However, the British physician Edward Jenner was credited in 1796 for bringing the vaccination to mainstream medical practice using cowpox inoculation against smallpox. Since then, vaccination has saved millions of lives affected by pathogens, including viruses such as smallpox, polio, influenza, rabies, and measles, just to name a few, and, most recently, the SARS-CoV-2. Among the variety of vaccines that have been developed and used are the conventional vaccines based on live-attenuated or killed viruses and the subunit vaccines containing one or more of the viral antigens. Considerations for their development include attenuation of pathogenicity, complete pathogen inactivation for safety, and preservation of potential epitopes for effective immunity [1,2]. Although conventional vaccines induce immune responses, they carry the risk of reversion to virulence, and environmental spread, or exhibit adverse effects on immunocompromised individuals. In recent times, conventional vaccines have faced significant challenges, including insufficient immune stimulation, in vivo instability, potential toxicity, and the requirement for multiple doses [2]. The subunit vaccines containing one or more of the viral antigens, on the other hand, are safer and can be readily produced at lower costs, but their efficacy is variable and they suffer from some of the above-mentioned challenges. To overcome these challenges and develop safer and more efficacious vaccines, nanotechnology platforms offer innovative solutions. The engineering of nanoparticles (NPs) as delivery systems for antigens can improve antigen stability, target specificity for immune cells, control antigen release kinetics, and be a potential gamechanger in vaccinology [3,4,5].

Several types of NPs have been employed in the field of vaccinology and targeted delivery of therapeutic agents. The inorganic NPs (INPs) can be designed to various shapes and sizes for optimal delivery and/or immunological responses. Although in some cases they exhibit adjuvant properties, they are often not biodegradable [6] and may exhibit toxicity. The organic NPs (ONPs) are derived from biomacromolecules, such as lipids, producing lipid NPs (LNPs) that can encapsulate nucleic acids encoding antigens, proteins, therapeutic drugs, etc.; proteins, generating protein NPs (PNPs) that can encapsulate and/or display antigenic proteins for immunological responses; and carbohydrates, with polymeric matrices such as poly-lactic-co-glycolic acid (PLGA)-forming NPs. Due to their biocompatibility, biodegradability, and low toxicity, the PNPs have been preferred over other NPs for a variety of applications including in vivo and in vitro molecular imaging, gene therapy, biosensing, targeted and non-targeted drug delivery, vaccine platforms, photodynamic and photothermal therapy, and MRI contrast agent [7,8,9,10,11,12].

Due to the availability of genomic sequence data for many organisms of prokaryotic and eukaryotic origin and powerful tools for genomic data mining, it has become relatively easier to identify and characterise naturally occurring PNPs with novel functions. For example, phylogenetic and bioinformatics analyses coupled with biochemical studies have resulted in the identification of over 900 potential encapsulin nanocompartments in prokaryotes, some of which play critical roles in iron mineralisation, oxidative and nitrosative stress resistance, and anaerobic ammonium oxidation [13]. However, it should be noted that although the existence of many PNPs in various domains of life has been predicted, only a few of them have been characterised structurally and functionally in greater detail. Some of these well-characterised PNPs are being used in various applications including imaging, diagnostics, targeted drug delivery, as well as vaccine platforms [7,8,9,14,15]. The use of naturally occurring proteins that form NPs and computationally designed synthetic proteins forming NPs are emerging as new players in vaccine technology. With the global health landscape continuously being impacted by emerging and reemerging pathogens causing epidemics and pandemics, nanotechnology-based vaccines will provide a rapid and adaptable platform to quickly respond to novel pathogen threats.

Here, we review the status of the protein-based nanotechnology approach in revolutionising vaccine development. Specifically, we emphasise the potential of naturally occurring proteins such as ferritin, lumazine synthase, and encapsulin, as well as other nanoparticles, including those derived from the capsid proteins of viruses, bacteriophages, and computationally designed synthetic protein-based NPs as emerging vaccine platforms for human and zoonotic viruses. These proteins serve as promising building blocks for the design and construction of advanced vaccine candidates. We highlight their suitability as carriers for antigens and as adjuvants through examination of their characteristics and immunogenic properties. Furthermore, we discuss the innovative methodologies employed to engineer these PNPs and explore their applications in enhancing vaccine efficacy, immunogenicity, and safety. As the field of nanotechnology continues to evolve, this review underscores the significant impact and potential future directions for utilising protein-based NPs to address the pressing challenges of vaccine development.

## 2. Protein-Based Nanoparticle (PNP) Vaccine Platforms: Classification, Advantages, and Limitations

PNPs occur in nature in living organisms such as bacteria, archaea, viruses, plants, insects, and mammals. They exist in different shapes including spherical, rod-shaped, disk-shaped, and in sizes ranging from 8 nm to 100 nm [15,16]. Importantly, they play many critical roles in biological processes including cellular homeostasis, storage, catalysis, protection of nucleic acids, and endocytic transport [15].

**Figure 1:**
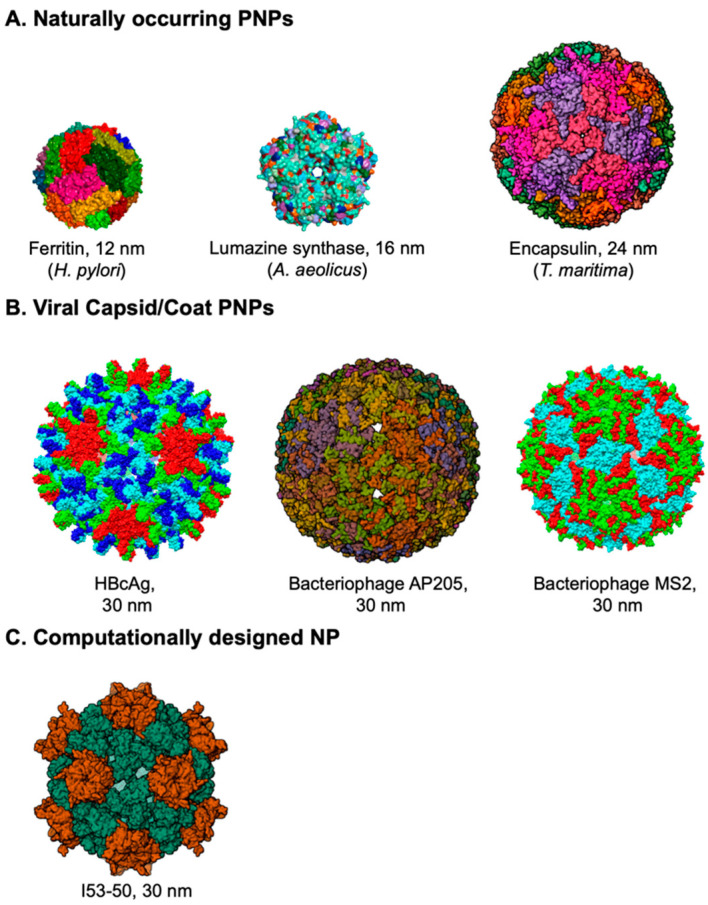
**Structures of some widely used protein nanoparticles.** (**A**) Naturally occurring PNPs. Surface structures of ferritin (PDB: 3BVE), lumazine synthase (PDB: 1HQK), and encapsulin (PDB: 3DKT). (**B**) Capsid/Coat PNPs of viruses and bacteriophages. Surface structures of hepatitis B core antigen, HBcAg (PDB: IQGT), AP205 (PDB: 5LQP), and MS2 (PDB: 2MS2). (**C**) Computationally designed NP. Surface structure of I53-50 (PDB: 6P6F). Ferritin, LS, HBcAg, and MS2 were generated using Chimera 1.17.3, while encapsulin, AP205, and I53-50 were retrieved from Protein Data Bank.

Based on their source, we classify the PNPs into three major groups. (1) Protein nanocages: Included in this group are the naturally occurring PNPs from nonviral sources such as ferritin from various prokaryotic and eukaryotic sources, lumazine synthase, encapsulin, E2p, and small heat shock proteins from prokaryotic sources. (2) The virus-like particles (VLPs): Included here are the PNPs from viral sources, generated with capsid proteins of viruses including hepatitis B virus (HBV), cucumber mosaic virus (CuMV), capsid or coat proteins of bacteriophages such as MS2, AP205, and Qβ. (3) Computationally designed PNPs, which include self-assembled PNPs based on modifications to naturally occurring proteins or de novo designed proteins such as mI3, I53-50. Figure 1 shows the structures of some of the representative members of each of the groups of PNPs that have been used as platforms for vaccines. Most PNPs are composed of many copies of self-assembling protein subunits from natural sources and are particulate in nature. The precise assembly of the individual protein subunits can result in a variety of well-characterised protein architectures such as fibers, rings, tubes, catenanes, knots, and cages [16], among which the protein cages or NPs have been widely used as vaccine platforms.

Compared to the soluble monomeric antigens, the PNP-based platforms displaying particulate antigens offer significant advantages [17], including the following. (i) They are of uniform shape and size, exhibit monodispersity, and display remarkable thermal and pH stability [18,19]; their spontaneous assembly and disassembly from individual subunits are highly reversible and reproducible [19], and they can be readily purified to near homogeneity using standardised protocols. (ii) The PNPs can be easily functionalised through genetic or chemical modifications. As the structures of many PNPs and their subunits have been solved at atomic resolution, they can be modified accordingly to generate stable structures with the presentation of antigens for optimal immune response. Modifications can be introduced at the surface, the interior, or at the subunit interface [20], which confer significant advantages for the use of PNPs as vaccine platforms. Due to modularity and precise locations at which the PNPs can be functionalised, they offer enormous advantages as a platform for the presentation of antigens to the immune system. (iii) They are biocompatible, biodegradable, and exhibit low toxicity. (iv) Like viruses and VLPs, the PNPs can be taken up by cells through the endocytic process, an important attribute for a vaccine platform. Unlike the soluble antigens, PNPs displaying antigens exhibit enhanced trafficking in lymph nodes, are efficiently internalised by antigen presenting cells (APCs), and are known to persist longer, which are also important factors for stronger humoral and cellular immune responses [21,22,23,24]. (v) Since our immune system responds efficiently to immunogens with sizes in the nanometer range [25,26], responses to PNPs displaying multiple copies of an antigen are expected to be far higher than those of a soluble antigen. (vi) As the repetitive array of an antigen improves immunogenicity and leads to enhanced immunological responses [27,28,29], the PNPs are desirable platforms for generating efficacious vaccines. PNP platforms deliver an ordered array of antigens that can result in stronger interactions with multiple B-cell receptors (BCRs), which is critical for downstream signaling for potent B-cell activation as well as antibody maturation [21,28,30,31]. (vii) PNPs smaller than 100 nm are readily taken up by peripheral or lymph node dendritic cells [32] for presentation of the antigen to trigger T cell immune responses [33,34]. (viii) Many PNP-based platforms display adjuvant properties [35,36,37,38,39], so vaccine delivery based on the PNP platform allows co-administration of the antigen, as well as the adjuvant simultaneously to the same immune cell for more robust immunological responses. (ix) Unlike the soluble antigens that can readily diffuse through the membranes of the blood endothelium and enter the blood circulation for faster systemic distribution, the particulate PNP-based vaccines have limited systemic distribution as they are transported in lymph to reach the lymph nodes. This can result in longer persistence and greater antigen levels in draining lymph nodes that may lead to better immune response and reduced toxicity [9,40].

**Figure 2:**
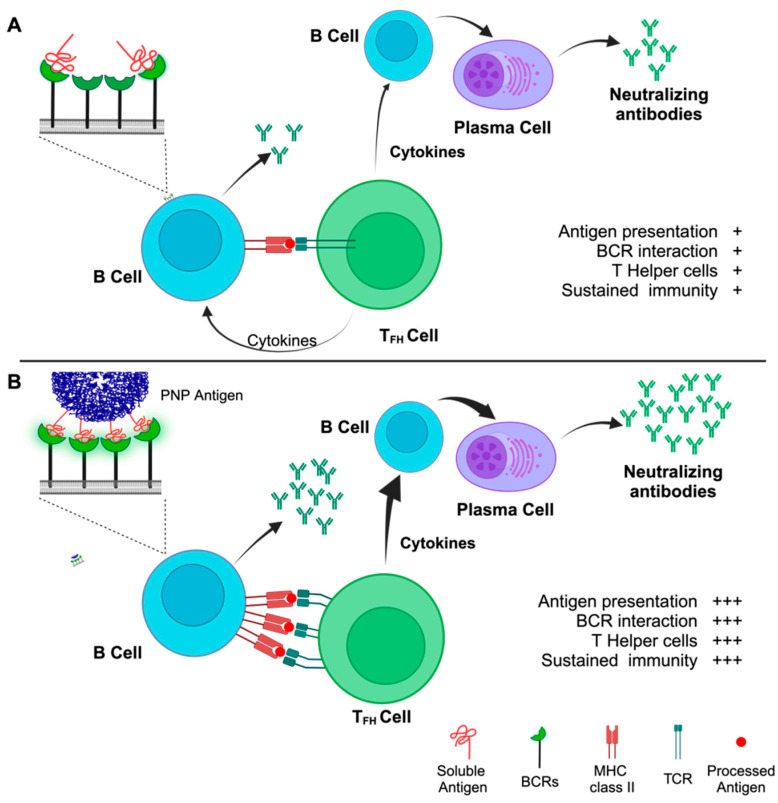
**The activation of humoral immune responses by soluble antigens vs. PNP antigens.** Soluble antigens interacting with BCRs (**A**) result in short-lived and less potent humoral immune response compared to PNPs displaying an ordered array of the antigen (**B**). Unlike soluble antigens, presentation of multiple copies of the antigen by a PNP leads to simultaneous interaction of multiple BCRs with the PNP (BCR clustering). This establishes a strong and durable antigen recognition by the B cell that translates to intracellular signaling, internalisation, and antigen processing for MHC class II presentation to T follicular helper (T_FH_) cells. This series of events evokes the secretion of regulatory cytokines by the T_FH_ cells that aid in the evolution of B cells to plasma cells, which produce antigen-specific neutralising antibodies. +, Low; +++, High. Figure created using BioRender.com.

Thus, PNP-based vaccine candidates displaying viral antigenic subunits can result in more efficient interaction with B-cell receptors (Figure 2), a crucial step in B-cell-induced immune responses that are multitudes of magnitudes higher than traditional vaccines. The suitability of PNP-based vaccines for large-scale production and ease of purification also add to the growing interest in this approach. Since PNPs are often assembled from one or two protein subunits, it is also possible to generate PNP-based vaccine candidates carrying a single antigenic determinant from one or multiple pathogens or antigens from multiple strains of a single pathogen. Such mosaic PNP-based vaccine candidates offer significant advantages over conventional candidates, as the need to prepare individual vaccines for each of the pathogens or strains can be avoided.

Although PNP-based vaccine platforms offer many advantages for enhanced vaccine efficacy, there are also challenges and potential limitations of their use. Attaching large antigens to a PNP subunit may result in inefficient assembly of the PNP due to steric hindrance, improper orientation, or misfolding of the antigen, leading to less ideal exposure or masking of appropriate epitopes for protective immunological responses. Genetic fusion of an antigen to the PNP platform may also result in the suboptimal level of expression [41]. Additionally, chemical conjugation or tag coupling to PNPs may result in incomplete decoration, a reduced number of antigens on the PNP surface, or conformational changes of the antigen, all of which would impact vaccine efficacy. Chemical conjugation or tag coupling approaches also require additional steps of process optimisation, assembly, and purification that can compromise the eventual yield of the desired vaccine candidate.

## 3. Approaches for Attaching Antigens to PNP Platforms

**Figure 3:**
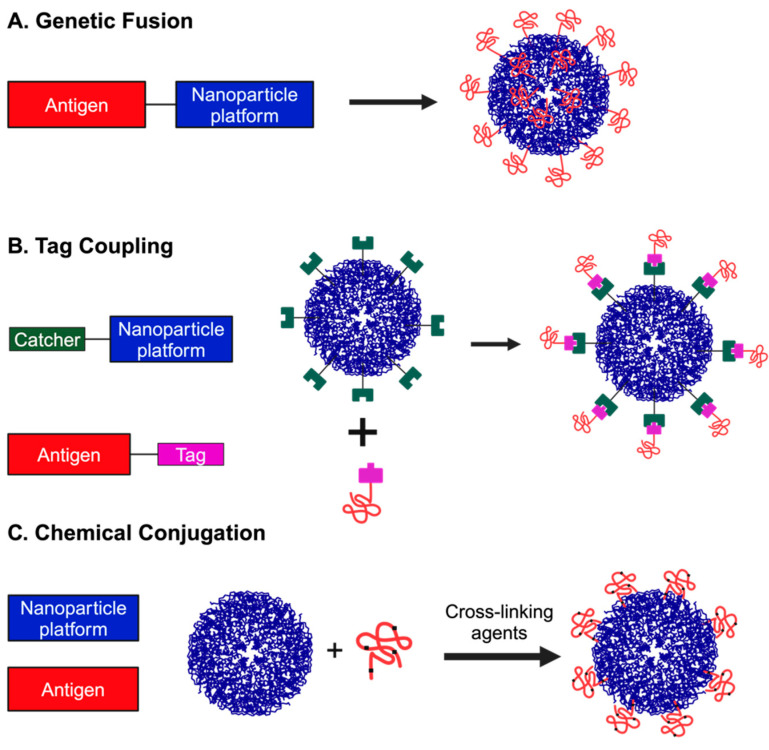
**Attachment strategies for PNPs and antigens.** (**A**) Genetic fusion of antigen with the PNP platform. Solid black line connecting both components represents a linker. (**B**) Tag coupling involves the genetic fusion of a receptor or catcher to one component and a tag to the other. This results in high-affinity interactions between tag and catcher leading to formation of PNP displaying the antigen on its surface. (**C**) Chemical conjugation makes use of chemical crosslinking agents to form irreversible bonds between reactive amino acid residues (cysteines, lysines, glutamates, and aspartates; represented by black dots on the antigen) in the antigen and the PNP. Figure created using BioRender.com.

Several different approaches have been adopted to attach antigens to PNP platforms (Figure 3). In the single-component system, the antigen is genetically fused with the sequences of the NP scaffold such that a single protein product is generated that assembles into a PNP carrying or displaying the antigen. In the two-component system, the surface of the NP is functionalised through genetic tags or chemical modifications for interaction with a correspondingly functionalised antigen. Each system has its own advantages and disadvantages. While in the single-component system, the ease of designing, generating, and purifying the PNPs adds significant advantages, the steric hindrance of large antigens may preclude or inhibit the assembly of the PNP for optimal immune response. In the two-component approach, while the steric hindrance is not a major constraint, the yield of tag-coupled or chemically modified PNP scaffold with the antigens could be compromised. Additionally, potentially incomplete, or uneven distribution of antigens on the PNP scaffold may also lead to variability in immune responses. However, all these approaches have been used for attaching antigens to PNP platforms.

### 3.1. Genetic Fusion

The simplest and most widely used approach to attach an antigen to a PNP platform is genetic fusion (Figure 3A). As PNPs are assembled from many copies of the same protein subunits, some of which have readily accessible amino and carboxy termini, it is relatively easy to modify the PNPs by genetically adding antigens. For example, the amino terminus of ferritin is exposed at the surface whereas its carboxy terminus is sequestered in the interior cavity of the NP. It is also possible to modify subunit interfaces if it does not interfere with NP assembly. The selected or designed antigen is fused in frame with the NP sequences. The antigen can be separated from the NP by flexible linker sequences to allow proper and efficient assembly of the PNP. In cells transfected with the plasmid encoding the antigen NP, the expressed fusion protein is assembled into PNPs, which can be purified through various biochemical methods. Challenges such as suboptimal level of expression of the fusion protein [41] have been addressed by a computational screening approach called Stabiliser for Protein Expression and Epitope Design (SPEEDesign) [42]. Difficulties with steric interference due to large size or the oligomeric nature of the antigen can be addressed using various tag coupling methods, discussed below. In silico computer modelling predictions can also be used for efficient self-assembly and stability of nanoparticles [43,44] for optimal exposure of antigens for vaccine design.

### 3.2. Tag Coupling

The tag-coupling approach involves adding a tag to an antigen and a catcher to the NP, expressing and purifying the two components separately, and mixing them together to generate the PNPs displaying the antigen (Figure 3B). Typically, the tag is genetically fused to one of the termini of the antigen and the catcher (or receptor) that selectively binds to the tag is fused to one of the termini of the NP such that the catcher is expressed on its surface. Following independent expression, purification, and mixing of the two components, the catcher associates with the tag with high affinity to generate the PNP displaying the antigen on its surface. The CnaB2 adhesin domain of the fibronectin-binding protein, FbaB of *Streptococcus pyogenes* is naturally stabilised by an isopeptide bond. The side chains of aspartic acid in a 13-residue peptide (SpyTag) and a lysine in its 116-residue protein partner (SpyCatcher) derived from the adhesin domain undergo spontaneous amidation, resulting in the formation of the covalent isopeptide bond [45]. This property has been exploited for use as a simple yet highly selective and robust approach to link antigens with PNPs. The SpyTag and SpyCatcher sequences can be fused to sequences of the antigen and PNP at either terminus. With the design of the more advanced SpyTag003–SpyCatcher003 possessing higher affinity with faster reaction kinetics [46], the SpyTag–SpyCatcher has become one of the most versatile and often used systems to attach an antigen to a PNP platform.

Other protein/peptide-based tag-coupling systems applicable for linking antigens to PNP platforms include SnoopTag/SnoopCatcher [47], sortase [48], and Barnase–Barstar [49]. The SnoopTag–SnoopCatcher is also based on isopeptide bond formation through a transamidation reaction between a lysine in a 12-residue peptide tag (SnoopTag) and an asparagine in a 112-residue cognate protein partner (SnoopCatcher) derived from an adhesin molecule from *Streptococcus pneumoniae*. The sortase A system involves peptide bond formation through a transpeptidation reaction between a sortase A recognition motif, LPXTG (X, any amino acid), and an oligoglycine sequence at the N-terminus of a protein, which is mediated by the enzyme sortase A from *Staphylococcus aureus*. The Barnase–Barnstar system, on the other hand, relies on strong noncovalent interaction between dimerisation domains of barnase, a 110-residue ribonuclease, and an 89-residue barnstar, a barnase inhibitor from *Bacillus amyloliquefaciens*. Additionally, the N- and C-termini of both barnase and barstar are away from their dimerisation domains and available for fusion [50]. Although the SpyTag–SpyCatcher system is widely used, it appears that other tag-coupling systems should also work well to allow specific assembly of the two components for the generation of the designed antigen–PNP complexes.

### 3.3. Chemical Conjugation

Chemical conjugation (Figure 3C) of an antigen to the surface of a PNP involves the treatment of the two components with crosslinking agents that generate highly stable irreversible bonds [51]. A variety of crosslinking agents that target surface-exposed cysteines, lysines, glutamates, and aspartates on the antigens and PNPs can be used [14]. However, chemical conjugation approaches are non-selective, can negatively impact the antigen or PNP structure, and lead to uneven decoration of the antigen, all of which could affect immune responses. In recent years, “click chemistry” has emerged as a popular approach in a variety of applications including protein labelling and modifications [52]. Among the four major classes of click reactions [53,54], the copper-catalysed azide–alkyne cycloaddition is widely used to link two protein components. Although these reactions are fast, highly selective, and efficient, they require additional steps of introducing the reactive functional groups into the antigen and the PNPs through the incorporation of amino acid analogs and unnatural amino acids into the proteins [55].

## 4. Vaccine Platforms Utilising Various PNPs

### 4.1. Protein Nanocages

#### 4.1.1. Ferritin

Ferritin is ubiquitous in all domains of life. It is a cytoplasmic iron storage protein with ferroxidase activity and is assembled as a NP, which stores iron within its hollow core. It also protects cells from the toxic effects of the Fenton reaction that generates hydroxyl radicals and reactive oxygen species [56,57,58]. The ferritin family consists of three distinct subfamilies: classical ferritins (or ferritins), heme-binding bacterioferritins, and DNA-binding proteins. While classical ferritins and bacterioferritins primarily serve in iron storage functions, the DNA-binding proteins facilitate iron detoxification [59,60,61]. Twenty four subunits of ferritin are assembled into a NP with octahedral symmetry that adopts a spherical cage-like structure [61,62,63] with inner and outer diameters of 8 and 12 nm, respectively (Table 1). The monomeric ferritin consists of five α-helices with the N-terminal helix being exposed outside and the C-terminal small helix sequestered inside of the hollow cavity of the NP [64]. The tertiary structure of ferritin is conserved across species. Vertebrate ferritin consists of two distinct subunits: the heavy (H) chain with a molecular mass of ~21 kDa and the light (L) chain with a mass of ~18 kDa. However, bacterial and plant ferritins exclusively comprise of only one subunit, aligning with the H chain of vertebrates [64].

In both mammalian and insect systems, ferritin NPs consist of 24 subunits of any combination of H and L chains [65] and are secreted from cells. On the other hand, bacterial ferritin NPs are assembled with 24 identical subunits into an octahedral configuration. Ferritin NPs exhibit remarkable thermal stability and resistance to chemical degradation [62,63]. These features have prompted investigators to delve extensively into exploring the structural and biochemical properties of ferritin across diverse organisms and examining its potential for developing a NP-based vaccine platform that leverages the distinctive characteristics of ferritin [9,66]. Central to the utilisation of ferritin as a vaccine platform is its capability to display antigens on the NP surface. Additionally, it has been shown not to induce any immune response [64].

Ferritin NP has emerged as an excellent platform for the development of viral vaccines since antigens can be displayed on the surface of the NP through multiple methods. In addition, the NPs possess excellent thermal and pH stability and can be readily assembled from monomeric subunits fused to antigens. Although ferritin from prokaryotic and eukaryotic sources have been used as platforms, the vast majority of studies (Table 2) have employed ferritin from *H. pylori*. The viral antigens are attached to the ferritin NP scaffold predominantly by genetic fusion at its N-terminus; however, tag coupling and chemical conjugation methods have also been used in some cases. The antigen–ferritin chimeric proteins are expressed in a variety of host cells including mammalian cells, insect cells, yeast, and bacteria. The expressed proteins assembled as PNPs can be designed to be secreted from the cells or extracted and purified for further characterisation and vaccination studies.

**Table 1:** Physical characteristics of some PNPs used in vaccine platforms.

Nanoparticle	Microorganism	Triangulation # (Protomers)	Size in nm	Reference
Ferritin	*H. pylori*	24	12	[61,63]
Lumazine synthase	*Aquifex aeolicus*	T = 1 (60)	15.4	[67,68]
	*Bacillus subtilis*	T = 3 (180)	29	[69]
Encapsulin	*Thermotoga maritima*	T = 1 (60)	24	[70]
	*Pyrococcus furiosus*	T = 3 (180)	31	[71]
	*Quasibacillus thermotolerance*	T = 4 (240)	42	[70]
E2p	*Geobacillus stearothermophilus*	T = 1 (60)	27	[72,73]
sHSP	*Methanococcus jannaschii*	24	12	[74]
HBcAg	Hepatitis B virus	T = 3/T = 4 (180/240)	28–32	[75]
CuMV_TT_	Cauliflower mosaic virus	T = 7 (420)	53.8	[76]
Qβ	Bacteriophage Qβ	T = 3 (180)	25	[77]
AP205 coat protein	Bacteriophage AP205	T = 3 (180)	25–30	[78]
MS2	Bacteriophage MS2	T = 3 (180)	27	[79]
I53-50	Synthetic	T = 2 (120)	30	[80]

**Table 2:** PNPs and their use in human virus vaccine development.

Nanoparticle	Pathogen	Antigen	References	Clinical Trials (Phase/NCT)
Protein Nanocages				
Ferritin	Influenza	Hemagglutinin (HA)/ Ectodomain of HA/ HA stem domain/HA-RBD	[81,82,83,84]	1/NCT03186781, 1/NCT03814720, 1/NCT04579250, 1/NCT5155319, 1/NCT01086657
	HIV	Envelope glycoprotein trimers/ ConM SOSIP trimer/ Native-like BG505 SOSIP trimers	[85,86,87,88]	1/NCT05903339
	HCV	sE2 protein	[89]	
	RSV	pre-fusion fusion (F)	[90]	
	ZIKV	E protein domain III (EDIII)	[91,92]	
	EBV	RBD of gp350/gH/gL or both gH/gL and gp42 glycoproteins	[93,94]	1/ NCT04645147, 1/NCT05683834
	SARS-CoV-2	Full-length spike (S) protein/S ectodomain with deletion of 70 C-terminal residues/RBD/RBD and heptad repeat (HR)/non-glycosylated RBD immunogen	[95,96,97,98,99,100,101,102]	1/NCT04784767, 1/NCT06147063
Lumazine synthase	HIV	Surface glycoprotein (gp120)	[85,103]	1/NCT05414786, 1/NCT03547245
	SARS CoV-2	S protein trimer/RBD/RBD-specific nanobodies obtained from a naïve alpaca phage display library	[102,104,105,106,107]	
	MERS-CoV	Multimeric RBD	[108]	
	Powassan virus	EDIII	[109]	
	Influenza	Ectodomain of the Matrix 2 (M2e)/mini-HA	[110,111]	
	EBV	gp350	[112]	
	Rift Valley fever virus	Head domain (Gn)	[113]	
Encapsulin	Influenza	M2e/HA	[114,115]	
	EBV	The domains I, II, and III of gp350	[93]	
	SARS CoV-2	RBD	[116]	
Encapsulin (EnDS)	SARS CoV-2	WA1 and BA.5 RBD	[117]	
**VLPs**				
E2p	HIV	B and T cell epitopes of HIV-1/ gp140	[85,118,119]	
	Ebola virus	GP	[120]	
	SARS-CoV-2	RBD	[102]	
HBcAg	ZIKV	EDIII	[121]	
	Influenza A	M2e	[122]	1/NCT00819013
CuMV_TT_	ZIKV	EDIII	[123]	
	SARS-CoV-2	RBD/RBM/fusion peptide	[124,125,126,127]	
Qβ VLPs	Influenza	M2e	[128]	
AP205	HIV	HIV Envelope protein	[129]	
	Influenza	HA (mosaic)	[130]	
	SARS CoV-2	RBD	[131,132]	3/NCT05329220
MS2	SARS-CoV-2	S protein	[133]	
Computationally Designed PNPs				
I53-50	RSV	F protein/trimeric DS-Cav1	[134,135,136,137]	1/NCT03049488
	Quadrivalent influenza vaccine candidate	HA trimers	[138]	1/NCT04896086, 3/NCT05007951
	SARS-CoV-2 RBD	RBD	[139]	3/NCT05007951
	HIV	SOSIP trimers	[140]	
	SARS CoV-2	Full-length S/Mosaic S/RBD/Mosaic RBD from multiple sarbecoviruses	[139,141,142,143,144]	1/2/ACTRN 12621000738820

The first use of ferritin NP as a vaccine platform was showcased by the presentation of influenza virus hemagglutinin (HA) in its native trimeric conformation on the NP surface. This resulted in significantly enhanced induction of neutralising antibodies, underscoring the potential of this vaccine candidate for heightened immune responses [81]. This approach has proven successful in creating a mosaic NP “universal influenza vaccine” containing HA from multiple subtypes, conferring heterotypic protection [82,83,145]. Studies have also shown that HA–ferritin NPs can stimulate prolonged germinal centre activity, indicative of an amplified and enduring immune response [84]. The heightened germinal centre response correlated with the maturation of memory B cells, thus enabling accelerated and more effective immune reactions upon subsequent exposures [84].

With the advent of the ferritin NP platform, significant progress was made in the development of HIV vaccines. Several vaccine candidates have now been generated using this platform (Table 2) and have been tested for their efficacy in animal models. The majority of the studies entailed the presentation of HIV-1 envelope glycoprotein trimers on the ferritin NP surface to augment immune responses. Robust humoral immune responses induced by the NP-based vaccine candidates with the involvement of germinal centres as compared to the use of soluble trimers were demonstrated [85,86,87,146,147]. Immunised animals showed a marked increase in the production of neutralising antibodies targeting various HIV-1 strains [87]. In an interesting study, ferritin NPs were tailored to present native-like BG505 SOSIP trimers, which were designed with the goal of recruiting V3-glycan-specific B cells by enhancing the accessibility of the V3-glycan patch epitope [88]. It revealed that animals immunised with these NPs exhibited elevated antibodies compared to their counterparts immunised with soluble trimers [87,147,148]. Interestingly, despite the heightened antibody response, the autologous 50% neutralisation titers against the Tier-2 BG505 virus in the ferritin-immunised rabbits did not exhibit improvement [87,148]. In contrast, rabbits immunised with ferritin nanoparticles carrying the consensus sequence-based ConM SOSIP trimer displayed enhanced neutralisation titers compared to those immunised with soluble ConM SOSIP trimers [86].

A hepatitis C virus (HCV) ferritin NP vaccine was engineered to address the challenges posed by the virus’ genetic diversity. Since the soluble E2 envelope protein (sE2) of HCV was shown to induce broadly neutralising antibodies against all HCV genotypes in mice and macaques [149], ferritin NP displaying the sE2 was found to elicit significantly higher neutralising antibody levels than sE2 in mice and neutralised all HCV serotypes [89]. In recent years, ferritin NPs have become the choice platform for generating vaccine candidates against many viral pathogens (Table 2). A vaccine candidate for the respiratory syncytial virus (RSV) displaying key neutralising epitopes and shielding the non-neutralising epitopes on the pre-fusion conformation of the fusion (F) glycoprotein was shown to induce durable antibody responses in nonhuman primates, generating potent neutralising antibodies both in vivo and in vitro [90]. Ferritin NP-based vaccine candidates were also recently developed for Zika virus (ZIKV). The envelope (E) protein domain III, which carries neutralising epitopes was displayed on either the human heavy chain ferritin [91] or *H. pylori* ferritin [92]. These vaccine candidates induced robust immune responses that protected animals from lethal ZIKV challenge. Furthermore, enhanced frequencies of interferon (IFN)-γ positive CD4 and CD8 T cells were also observed, indicating the induction of both humoral and cell-mediated immune responses [92].

Using a hybrid *H. pylori*-bullfrog ferritin as a platform, investigators displayed a functionally conserved receptor-binding domain of gp350 of Epstein–Barr virus (EBV). This strategy induced potent neutralising antibodies in both mice models and non-human primates (NHPs) that were 10- to 100-fold higher than the soluble gp350 [93]. Likewise, in a recent study, ferritin NPs displaying the fusion components of EBV, namely the glycoprotein H (gH) and gL or gH/gL and gp42 were shown to induce robust immune responses in mice and NHPs that could neutralise the virus in B cells and epithelial cells [94]. These studies underscore the potential of ferritin-based nanoparticle strategies in augmenting the efficacy of vaccines against challenging viruses like EBV.

With the onset of the SARS-CoV-2 pandemic and the urgent need for vaccines, attempts were made to use the PNP-based platforms including the ferritin NP-based system to generate several vaccine candidates against the virus (Table 2), some of which have moved to clinical trials [41,139,150]. The ferritin NP-based candidates display either the full-length spike (S) protein [95,96,97,104], the full S ectodomain [151], the S ectodomain with deletion of 70 C-terminal residues [98], the receptor-binding domain (RBD) [96,99,100,101,152,153,154,155,156], RBD and heptad repeat (HR) [100], or a non-glycosylated RBD immunogen generated with SPEEDesign computational approach that incorporated deep mutational scanning data [102]. The majority of these candidates were generated via tag coupling or genetic fusion approaches. In various animal models and virus challenge studies, these vaccine candidates induced robust and sustained neutralising antibody responses and cellular immune responses that conferred protection against virus challenge.

#### 4.1.2. Lumazine Synthase

Lumazine synthase (LS), a naturally occurring protein in bacteria and archaea, fungi, and plants, is integral to riboflavin (vitamin B2) synthesis and holds significant promise in the field of vaccine design [157]. LS is composed of 60 identical subunits arranged in 12 pentameric units forming an icosahedral NP structure of 15.4 nm and 9 nm outer and inner diameters, respectively [67,68]. Interestingly, LS from *Bacillus subtilis* at high pH conditions can reassemble into a larger NP of about 29 nm containing 180 subunits [69]. Both the N- and C-termini of LS are exposed on the surface of NP and exhibit threefold and fivefold symmetry, with the N-terminus appearing closer to the threefold apex compared to the C-terminus. This proximity of the termini to the symmetry axis has significant implications for stabilising the presentation of trimeric or pentameric antigens in an orderly array.

By genetically fusing through a coiled-coil linker at the C-terminus of LS from *Aquifex aeolicus*, a rationally designed gp120 was shown to induce a robust humoral response in mice compared to the antigen presented without the NP platform [103]. Furthermore, the designed vaccine candidate activated the germline and mature VRC01-class B cells. Presentation of native-like trimeric HIV-1 gp120 or gp140 that display 20 spikes on LS NP surface led to robust stimulation of B cells carrying cognate VRC01 receptor [85]. The LS NP platform was also used to generate HIV-1 vaccine candidates targeting the germinal centres for robust broadly neutralising humoral immune responses [147,158].

Building on these successes, the trimeric S protein of SARS-CoV-2 presented on the surface of LS NP was shown to elicit a significantly higher neutralising antibody response compared to the S protein without the NP platform. In similar studies, the SARS-CoV-2 RBD displayed on the surface of LS NP was demonstrated to induce potent and long-lasting neutralising antibody responses that conferred near complete protection of animals against the virus challenge [102,105]. Importantly, the neutralising antibodies generated by this vaccine candidate could neutralise not only several variants of SARS-CoV-2 but also SARS-CoV-1 and its related bat coronaviruses [105]. In a different approach, SARS-CoV-2 RBD was linked to LS NP from *Brucella abortus* via sortase A-mediated transpeptidation reaction. This yielded a variable number of RBD molecules ranging from as high as 6–7 to as low as 1–2 per decamer of LS. The humoral responses in vaccinated animals were significantly higher with LS NP carrying a higher number of RBD molecules compared to those with fewer RBD molecules [106]. In an interesting recent study, the LS NP platform was used to present RBD-specific nanobodies obtained from a naïve alpaca phage display library. These nanobodies on LS NP were found to bind and neutralise pseudotyped SARS-CoV-2 efficiently. The study highlights LS’s role in enhancing nanobody efficacy against SARS-CoV-2 variants [107].

The LS NP platform has been used in recent years to develop other viral vaccine candidates including MERS-CoV RBD [108], Powassan virus envelope protein domain III [109], pseudorabies virus glycoprotein D [159], IAV mini-HA, and extracellular domain of M2 [110,111], EBV gp350 [112], and the head domain (Gn, a target of neutralising antibodies) of Rift Valley fever virus glycoprotein [113]. Genetic fusion or tag coupling was used to link the viral antigens to the NP scaffold. Vaccination with these candidates has elicited robust and high-quality antibody responses, including effective neutralisation and establishment of mucosal immunity compared to monomeric antigen, and conferred protection in animal models, thus establishing that LS is a desirable PNP platform for the development of vaccine candidates against viral pathogens.

#### 4.1.3. Encapsulin

Encapsulins are widely distributed in bacteria and archaea and play important roles in iron storage and mineralisation, oxidative and nitrosative stress resistance, and anaerobic ammonium oxidation [13,160]. Recently, they have been identified as a class of prokaryotic nanocompartments (NCs) or NPs and have garnered significant interest in various fields of biology, including biomedicine and nanotechnology. Encapsulin self-assembles into NCs generating icosahedral structures with diameters ranging from 25 to 42 nm. Their remarkable ability to encapsulate cargo proteins with specific carboxy-terminal residues that bind to the internal surface of the NCs makes them particularly intriguing for NP vaccine design [70,160]. Encapsulin also contains a flexible loop on the surface of the NCs/NPs, which can be exploited to insert peptides or antigens for their surface display [7]. Encapsulin NCs exhibit variability in size and subunit composition depending on their origin. For instance, *Pyrococcus furiosus* encapsulin NC is made up of 180 protomers [71] with a diameter of 30–32 nm. In contrast, encapsulin NC from *Thermotoga maritima* consists of 60 protomers, measuring 24 nm in diameter [70], while that of *Quasibacillus thermotolerance* can assemble with up to 240 protomers, resulting in a larger diameter of 42 nm [70].

Because of the presence of a flexible loop on the surface and its cargo-loading ability, encapsulin NCs/NPs can be used for the simultaneous display of an antigen on the surface, as well as the incorporation of a second antigen into the interior of the cavity for the rational design of vaccines. In a proof-of-concept study, the ectodomain of the M2 protein of IAV was inserted in the surface loop and GFP was fused to the carboxy terminus of encapsulin from *T. maritima* [114]. This vaccine design resulted in elicitation of antibody responses to both antigens in vaccinated animals. In another study, an encapsulin NC displaying on its surface a conserved HA stem domain of IAV was shown to confer protection against related IAV strains [115]. To enhance vaccine effectiveness against EBV, the domains I, II, and III of gp350 were fused at the C-terminus of encapsulin, enabling the antigen on the NP surface [93]. The encapsulin NP vaccine induced potent neutralising antibodies in mice and NHPs that were up to 100-fold higher than the soluble gp350 [93]. To use this NP platform for SARS-CoV-2 vaccine development, an encapsulin–RBD was designed, which exhibited exceptional antigenicity and long-term stability. In mouse models, the vaccine elicited robust neutralising antibody responses following two immunisations, effectively neutralising both the wild-type virus and its alpha, beta, and delta VOCs [116]. Even a single dose of the vaccine induced substantial neutralisation activity against the omicron variant, despite reduced sensitivity compared to other variants. In a recent study, a stabilised version of encapsulin (EnDS) NP was created by introducing disulfide bonds between protomers [161]. Using the EnDS, the RBDs of SARS-CoV-2 isolates from WA1 and BA.5 carrying RBD-stabilising mutations were displayed via the spyTag/SpyCatcher system to generate individual or mosaic NPs. The NPs were shown to induce significantly higher homologous and heterologous neutralising titers [117] in mice. Interestingly, the study revealed that these vaccines can also elicit higher neutralising antibody titers against other β-coronaviruses [117].

#### 4.1.4. Other Nanocages

Several other PNPs have also been used as vaccine platforms in recent years. The E2p (dihydrolipoamide acetyltransferase) from *Geobacillus stearothermophilus*, a component of the pyruvate dehydrogenase complex, assembles as a 27 nm NP with icosahedral symmetry consisting of 60 subunits [72,73]. The E2p NPs displaying a variety of antigens including B and T cell epitopes of HIV-1 antigens through genetic fusion have resulted in the induction of humoral as well as potent helper and cytotoxic T cell responses [118,119,162,163]. Recently, presentation of stabilised gp140 trimer on the surface of E2p NP led to the stimulation of B cells carrying the cognate receptor [85]. Ebola virus GP rationally designed to form trimers when presented on the surface of E2p elicited vaccine-induced B-cell responses and generated cross-ebolavirus neutralising antibodies, suggesting a promising vaccine strategy for filoviruses [120]. When an enhanced SARS-CoV-2 RBD immunogen was displayed on E2p NP, elicitation of potent neutralising antibodies was demonstrated in NHPs [102]. Although E2p NP has not been as widely used as the other NPs discussed above, it holds significant promise since foreign antigens fused to the N-terminus of the E2p core can be readily displayed on the NP surface for immune responses. Another interesting protein is the small heat shock protein (sHSP) from *Methanococcus jannaschii*, which confers thermotolerance to the organism. Twenty-four subunits of sHSP are assembled into a hollow spherical NP of 12 nm in the outer and 6.5 nm in the inner diameter [74]. Interestingly, mice treated with sHSP NP alone (without any specific viral antigen) were protected against several respiratory viruses [164], indicating that the NP platform itself confers protection. Furthermore, the exterior and interior of the NP can be modified by genetic and chemical methods [165], suggesting that sHSP NP can be a highly versatile platform for vaccines.

### 4.2. VLPs

The capsid proteins of many viruses such as hepatitis B virus (HBV), cucumber mosaic virus (CuMV), cowpea chlorotic mottle virus, and the coat proteins of bacteriophages such as AP205, MS2, and Qβ are assembled into PNPs having various sizes and symmetry (Table 1) in the absence of their nucleic acid genome. These PNPs can also be readily functionalised by genetic fusion, tag coupling, or chemical modifications to display foreign peptides or proteins on their surface without compromising the PNP assembling capabilities. These attributes have been exploited in recent years for their use as vaccine platforms [12]. The hepatitis B virus core antigen (HBcAg) is assembled as two different types of icosahedral particles consisting of 180 (90 dimers; 26 nm; T = 3) or 240 (120 dimers; 30 nm; T = 4) subunits [75]. Foreign proteins can be displayed on the HBcAg PNP surface readily by genetic fusion at the major immunodominant epitope (MIE), the N- or the C-termini [166,167]. The E protein domain III of ZIKV (zE-DIII), when displayed on the surface of HBcAg through genetic fusion at its C-terminus, induced robust humoral and cellular immune responses in mice, and conferred protection from multiple ZIKV strains. HBcAg PNP appears to be a very exciting platform as it can be genetically modified at many locations for surface display of an antigen and the observation that it is known to contain strong T-cell epitopes [121].

Another platform that has gained significant attention is the cauliflower mosaic virus capsid PNP [76] incorporating a universal T-cell epitope from tetanus toxin (CuMV_TT_) [168]. Using this platform, the ZIKV E-DIII, displayed on the surface of the NP through chemical crosslinking was shown to elicit neutralising antibody response [123]. The same platform was used to generate several SARS-CoV-2 vaccine candidates using the RBD [124,125], receptor-binding motif alone (RBM) [126], or along with fusion peptide [127]. The antigens were either genetically fused or chemically crosslinked to the PNP platform. These studies revealed that the antigens are highly immunogenic in animals, induce neutralising antibody responses that are cross-reactive, and can potently neutralise the virus under in vitro conditions.

In addition to the viral capsid PNPs, the coat proteins of bacteriophages that generate highly ordered spherical structures with differing triangulation numbers and symmetry have also been used as platforms for vaccine development. The Qβ VLPs [77] when linked to the extracellular domain of the M2 protein of influenza virus, the resulting VLP induced strong M2-specific antibody responses and protected animals against lethal challenge with influenza virus [128]. The coat protein of bacteriophage AP205 [78] can generate stable PNPs and its N- and C-termini can accept insertions of antigens without adverse effects on PNP integrity or stability [169]. Tag coupling can also be used to display antigens on the PNP surface. This platform was used to display the trimeric envelope protein of HIV-1 via the SpyTag/SpyCatcher coupling. Immunisation of mice, rabbits, and NHPs led to the elicitation of broadly neutralising antibodies [129]. A similar approach was used to generate a vaccine candidate for IAV by conjugating up to eight different homotypic or heterotypic (mosaic) trimers of HAs via the SpyTag/SpyCatcher system. Immunisation of mice with these vaccines resulted in cross-reactive antibody responses [130]. The SARS-CoV-2 RBM or RBD was displayed on the surface of the AP205 coat protein PNPs through genetic fusion at its C-terminus [131] or via SpyTag/SpyCatcher coupling [132]. Mice administered with these vaccine candidates induced elevated levels of serum antibodies and significant levels of neutralising antibody titers. These studies suggest that the AP205 coat protein PNP platform is a promising platform for vaccine development. The capsid of the bacteriophage MS2 has an icosahedral symmetry with 180 subunits of its coat protein arranged as 90 homodimers. A genetically fused single-chain dimer of two identical coat proteins can also assemble efficiently and can accommodate peptides in a surface-exposed loop region [79]. Taking advantage of this, the full-length S protein of SARS-CoV-2 was displayed on the surface of the PNP by biotin–streptavidin tag coupling [133]. Following immunisation, the nanoparticles were shown to generate high titers of neutralising antibodies and protected animals from the SARS-CoV-2 challenge, suggesting that this platform could be used for other pathogens.

### 4.3. Computationally Designed PNPs

Computationally designed NP assemblies have gained significant advancement since the development of Rosetta3 for modelling symmetrical protein structures [170]. This has led to the first design of a highly stable 25 nm in size icosahedral protein structure (mI3) from 60 subunits of 2-keto-3-deoxy-6-phosphogluconate (KDPG) aldolase from *T. maritima* [171]. Subsequent studies led to the design and characterisation of a novel icosahedral particle (I53-50) of 30 nm consisting of 20 trimeric (A component) and 12 pentameric (B component) building blocks for a total of 120 subunits [80]. This I53-50 PNP has become the preferred choice as a platform for vaccine development.

Using the I53-50 platform, a promising vaccine targeting the fusion (F) protein of the respiratory syncytial virus was developed. Traditional vaccine candidates focused on the more stable post-fusion structure due to the instability of the prefusion conformation, yielding limited efficacy in clinical trials [134,135,136]. However, employing a structure-guided approach, a self-assembling PNP displaying 20 copies of trimeric DS-Cav1, a prefusion-stabilised F protein variant, was generated. This PNP vaccine induced 10-fold greater neutralising antibody titers compared to soluble DS-Cav1 in mice and NHPs [137], demonstrating the significant potential of this I53-50 PNP platform for other viral pathogens with trimeric surface glycoprotein targets. Using a similar approach, the HA trimers of the licensed quadrivalent influenza vaccines displayed on the I53-50 platform induced broadly protective antibody responses at similar or higher levels than the licensed quadrivalent vaccines [138].

The I53-50 PNP platform was employed to enhance the immunogenicity of HIV-1 envelope trimers with the goal of inducing broadly neutralising antibodies that can neutralise both neutralisation-sensitive and neutralisation-resistant viruses. To achieve this, a soluble HIV-1 envelope trimer, stabilised SOSIP trimers, expressing multiple epitopes for broadly neutralising activity [172], was presented as 20 trimers on the surface of I53-50 PNP. This PNP vaccine candidate was shown to increase immunogenicity and enhance the quality of antibody response upon immunisation [140], indicating that the I53-50 PNP would be a suitable platform for advancing efficacious HIV vaccine development. In line with these goals, further studies using this platform revealed that such a vaccine candidate could generate substantial enhancement of neutralising antibody titers compared to the soluble SOSIP trimers in rabbit immunisation experiments [173]. Further, the neutralising responses were directed to an immunodominant epitope, suggesting that this PNP has the potential to be an excellent platform for HIV vaccine development.

With the onset of the SARS-CoV-2 pandemic, the mI3 and I53-50 PNP became the platforms of choice to quickly generate a variety of vaccine candidates against the virus. Using the mI3 platform, homotypic PNPs displaying the RBD from only the SARS-CoV-2 or mosaic PNPs displaying RBDs from four or eight different betacoronaviruses were shown to induce antibodies not only to the SARS-CoV-2 RBD but also to the RBDs of other viruses equally well [174]. Additionally, the mosaic nanoparticles induced antibodies that could also recognise mismatched virus strains [174]. The ease with which the two components of I53-50 could be readily constructed, produced, purified, and assembled into PNPs displaying the SARS-CoV-2 antigenic determinants accelerated the development of several excellent vaccine candidates. In these studies, either the full-length S [142], mosaic S from several variants of concern [143], the RBD [139,141], or the mosaic RBD from multiple sarbecoviruses [144] was displayed as trimeric subunits on the surface of the I53-50 through genetic fusion or tag coupling. In various animal models, these vaccine candidates induced significantly higher neutralising antibody responses compared to their soluble trimeric counterparts. These studies suggest that I53-50 is an excellent antigen display platform for vaccine development.

## 5. Summary, Challenges, and Future Prospects

As emerging and re-emerging viral and other infectious agents pose serious health challenges worldwide, the rapid development of safe, highly efficacious, and cost-effective vaccines is needed. PNP-based vaccine platforms offer significant advantages in this regard. These platforms are highly reproducible and scalable, and the target antigen can be attached readily through a variety of approaches without significantly compromising its native-like structure and exhibit adjuvant-like properties for enhanced immune responses and protection. In recent years, many PNPs have been used as excellent platforms for displaying viral antigens and such vaccine candidates have unequivocally shown to induce significantly elevated immune responses resulting in protection from the pathogen. With the ability to computationally design and experimentally characterise icosahedral PNPs, it will be possible to further optimise these structures with regard to their primary sequence, subunit interaction, assembly, and presentation of antigens. Such optimisations would undoubtedly lead to candidate vaccines with unprecedented safely and efficacy.

Although the PNP platforms have been used in recent years for the development of vaccines, challenges remain to be addressed in terms of their utility from preclinical laboratory studies to widespread clinical applications in humans. One of the major challenges is the immune response against the vaccine platform itself, which could lead to rapid clearance of the vaccine and reduced therapeutic efficacy [175]. By comparing several genetic fusions of SARS-CoV-2 RBD to different PNPs, such as those derived from ferritin, lumazine synthase, foldon, and β-annulus, it was recently shown that while the β-annulus platform induced robust virus neutralisation and T cell responses, the minimal immune response was triggered against the platform itself compared to the other platforms [176]. Therefore, identification and further in-depth studies of PNP platforms for optimal immune responses against the viral antigen while minimising the immune responses against the platform are of utmost priority. In this regard, PNP vaccine candidates with different platforms could also be used for booster injections, if needed, to avoid vaccine clearance and reduced efficacy. Since PNPs are readily cleared from the body, another challenge is to ensure the in vivo bioavailability of the PNP vaccine candidates for optimal immune responses and vaccine efficacy. In this regard, further studies to optimise conditions to coat PNP surfaces with compounds such as polyethylene glycol (PEG) to reduce their clearance and enhance vaccine efficacy [177] are needed. Although much has been learnt regarding the physical characteristics of PNPs for immune responses [17,44], further studies, through computational advances to generate optimal PNP platforms with respect to size, shape, surface charges, antigen insertion sites, and distance between displayed antigens, need to be conducted to establish most optimal structures for robust immune responses. Other challenges such as safety profiles, manufacturing, scale-up production, and reproducibility also need to be worked out to make a jump from preclinical development to clinical deployment of PNP-based vaccines.

Despite these challenges, the PNPs offer significant promise for rapid and efficient development of vaccine candidates against emerging and reemerging viral threats. With the development of algorithms for accurate prediction of protein structures using AlphaFold [178] and RoseTTAFold [179], the future of PNP-based vaccine design and their use in clinical settings appear bright.
